# An Introduction to the Study of Human Anatomy

**Published:** 1834-04-01

**Authors:** 


*An Introduction to the Study of Human Anatomy.* By James Paxton, m.r.c.s. &c. In Two Volumes. Vol. II.—London, 1834. 8vo. pp. 370.

This elementary system of anatomy will be useful to beginners, but is chiefly intended, we believe, for non-professional persons. The woodcuts, which are very numerous, will recall to the former class what they have already seen, and to the latter will often be a substitute for what they may never see. It would be absurd to give many extracts from a work of this kind, and we shall therefore content ourselves with a single one relating to a subject which, though treated of by several great writers, is not yet so familiar to every one as it ought to be; we mean *muscular sensation.*

"To the sensitive department of the fifth pair, and the compound spinal nerves, is assigned muscular sensation. This is the sixth sense. All our conceptions of weight and resistance, and motion in general, are derived from our muscles. The muscular system then may be considered a distinct organ of sense as well as motion; each motion of the invisible muscles is accompanied with a certain feeling, which may indeed be complex, as arising from various muscles, but which is considered by the mind as one, and it is this peculiar feeling, attending the action of the muscular fibres, which we distinguish from every other sensation. To exemplify this, I might refer to the state of the muscles in cramp of the limbs, and in rheumatic affections; in such morbid conditions their structure becomes painfully sensible. But let us call to mind the phenomenon which every one must have experienced, I mean the feeling of fatigue; this is a muscular sensation: a sensation of which the muscles are the organs, as much so as that of the eye and the ear are the organs of sight and hearing. Every bodily effort depends on muscular contraction; and long and frequent contractions, that is, continued exercise, occasion a peculiar uneasiness which demand repose. Powerful and protracted exertions produce painful sensations to the muscular sense: a more moderate degree of exercise is attended with agreeable sensations. With a healthy state of body, there is a muscular pleasure in exertion. Thus, the child who is not playful, is not healthy. There is a muscular gratification, if I may so express myself, in every limb, in the games and pastimes of the schoolboy.

"Dr. Brown, without being aware that there was a peculiar set of nerves appropriated to muscular sensation, observes, that 'nature in the other animals, whose sources of general pleasure are more limited, has converted their muscular system into an organ of delight. It is not in search of richer pasture that the horse gallops over the field, or the goat leaps from rock to rock; it is for the luxury of the exercise itself. It is this appearance of active life which spreads a charm over every little group with which the Deity animates the scenery of nature.' We may therefore consider that the muscular system is not merely the living machinery of motion, but that it is also truly an organ of sense." (P. 177.)

